# *Argyrella richardsiae*, a new species of Melastomataceae from the wet miombo woodlands of south-central Africa

**DOI:** 10.3897/phytokeys.82.12914

**Published:** 2017-07-03

**Authors:** Marie Claire Veranso-Libalah, Robert Douglas Stone, Gudrun Kadereit

**Affiliations:** 1 Department of Botany and Plant Physiology, University of Buea, P.O. Box 63, Buea, Cameroon; 2 School of Life Sciences, University of KwaZulu-Natal, Private Bag X01, Pietermaritzburg 3209, South Africa; 3 Institut für Molekulare Physiologie, Johannes Gutenberg-Universität Mainz, D-55099 Mainz, Germany; 4 Institut für Organismische und Molekulare Evolutionsbiologie, Johannes Gutenberg-Universität Mainz, D-55099 Mainz, Germany; 5 Marie Claire Veranso-Libalah

**Keywords:** Africa, Angola, *Argyrella*, *Dissotis*, *Heterotis*, Melastomataceae, new species, Tanzania

## Abstract

A new species from the wet miombo woodlands of Tanzania and Angola, *Argyrella richardsiae* Veranso-Libalah & G.Kadereit, **sp. nov**. (Melastomataceae, Melastomateae), is described and illustrated. Although the widespread *Argyrella canescens* also occurs in Tanzania and northeastern Angola, *A. richardsiae* is morphologically most similar to *Argyrella bambutorum* known only from the Northwest of Cameroon, but differs by its indumentum of glandular trichomes on the entire plant (versus a mixture of stellate and glandular trichomes in other species of *Argyrella*), leaf-blades with serrulate margins (versus entire margins in *A. bambutorum*) and lateral nerves that become faint mid-way and never reach the leaf apex (versus conspicuous lateral nerves percurrent from the base to the apex in *A. bambutorum*). A preliminary conservation status of Endangered (EN) is proposed for *A. richardsiae* following the IUCN Red List Categories and Criteria. A taxonomic key and distribution map of all *Argyrella* species is also included.

## Introduction

*Argyrella* Naudin was first described by Naudin (1850) and later treated by Triana (1872) as Dissotis sect. Argyrella (Naudin) Triana. Later, Fernandes and Fernandes (1969, 1970) transferred the type species *D. canescens* (E.Mey. ex Graham) Hook.f. as well as *D. angolensis* Cogn. to Dissotis subgen. Argyrella (Naudin) A.Fern. & R.Fern. These two species together with four other *Dissotis* species previously treated in “séries des *Dissotis* canescents” by Jacques-Félix (1953) were then transferred to Heterotis Benth. sect. Argyrella (Naudin) Jacq.-Fél. by Jacques-Félix (1981, 1995).

A recent molecular phylogenetic analysis of African Melastomateae by Veranso-Libalah et al. (2017) included four species previously treated in Heterotis sect. Argyrella [*H. canescens* (E.Mey. ex Graham) Jacq.-Fél., Heterotis angolensis (Cogn.) Jacq.-Fél. var. bambutorum (Gilg & Ledermann ex Engl.) Jacq.-Fél., *H. amplexicaulis* (Jacq.-Fél.) Aké Assi and *Agyrella* sp.]. Although the phylogenetic relationships between these species were not well-resolved, all four species formed a well-supported clade in the Bayesian inference, maximum likelihood and parsimony analyses. In addition, their study of herbarium material morphologically supported the resurrection and updated circumscription of *Argyrella* with six species including five new combinations. *Argyrella* together with *Guyonia* Naudin, *Melastomastrum* Naudin, *Anaheterotis* Veranso-Libalah & G.Kadereit and *Tristemma* Juss. belong to the ‘Pseudoheterotis’ clade which consists mainly of herbs with persistent calyx lobes lacking intersepalar appendages. *Argyrella* is closely related to the monospecific *Anaheterotis* but is distinguished by having stellate and/or glandular trichomes on the entire plant (glabrous in *Anaheterotis*), entire to serrulate leaf margins (versus densely serrate margins ending in prominent ciliate trichomes in *Anaheterotis*). Also, *Argyrella* can be distinguished from *Heterotis* by its erect growth (versus a decumbent habit in *Heterotis*), calyx-tubes with an indumentum of stellate and/or glandular trichomes [versus stalked stellate emergences in *Heterotis* (except in *H. decumbens* (P.Beauv.) Jacq.-Fél. which has simple trichomes)], and paniculate inflorescences (versus flowers solitary or in cymes in *Heterotis*). The chromosome number *n* = 17 in *A. canescens* (E.Mey. ex Graham) Harv. and *A. amplexicaulis* (Jacq.-Fél.) Veranso-Libalah & G.Kadereit is the same as those counted in species of *Melastomastrum* and *Tristemma* (Favarger 1952, 1962).

From our comparative study of herbarium collections through visits (BR and BRLU), loans (BR, BRLU, C, EA, MO, NHN, KEW and UPS), and online repositories: BM (http://data.nhm.ac.uk/), P (https://science.mnhn.fr/all/search), LISC (http://actd.iict.pt/) and COI (https://www.uc.pt/herbario_digital/catalogues), we have identified a wet miombo woodland species of *Argyrella* from Tanzania and Angola that is new to science, described and illustrated herein. All measurements were taken from dried specimens. The extent of occurrence (EOO) and area of occupancy (AOO) were calculated using GeoCAT (Bachman et al. 2011), and a preliminary conservation status is proposed following the IUCN Red List Categories and Criteria (IUCN 2012). We also provide an identification key and a distribution map of the seven species presently recognised in *Argyrella*.

## Results

### Argyrella richardsiae

Taxon classificationPlantaeMyrtalesMelastomataceae

Veranso-Libalah & G.Kadereit sp. nov.

urn:lsid:ipni.org:names:77163878-1

Figure 1

#### Type.

TANZANIA. Katavi region: Mpanda district, 19 km on Mpanda-Uvinza road, seepage areas in tall *Julbernardia paniculata*, *Terminalia mollis* woodland, grey and sandy soils, 6°14'S, 30°59'E, 1100 m, 14 May 1997 (fl & fr) , *S. Bidgood, D. Sitoni, K. Vollesen & C. Whitehouse 3935* (Holotype: K! [K000771858!]; isotypes: K! [K000771858!]; BR! [BR0000013189358!], C!, EA!, P! [P05222349!]).

**Figure 1.**
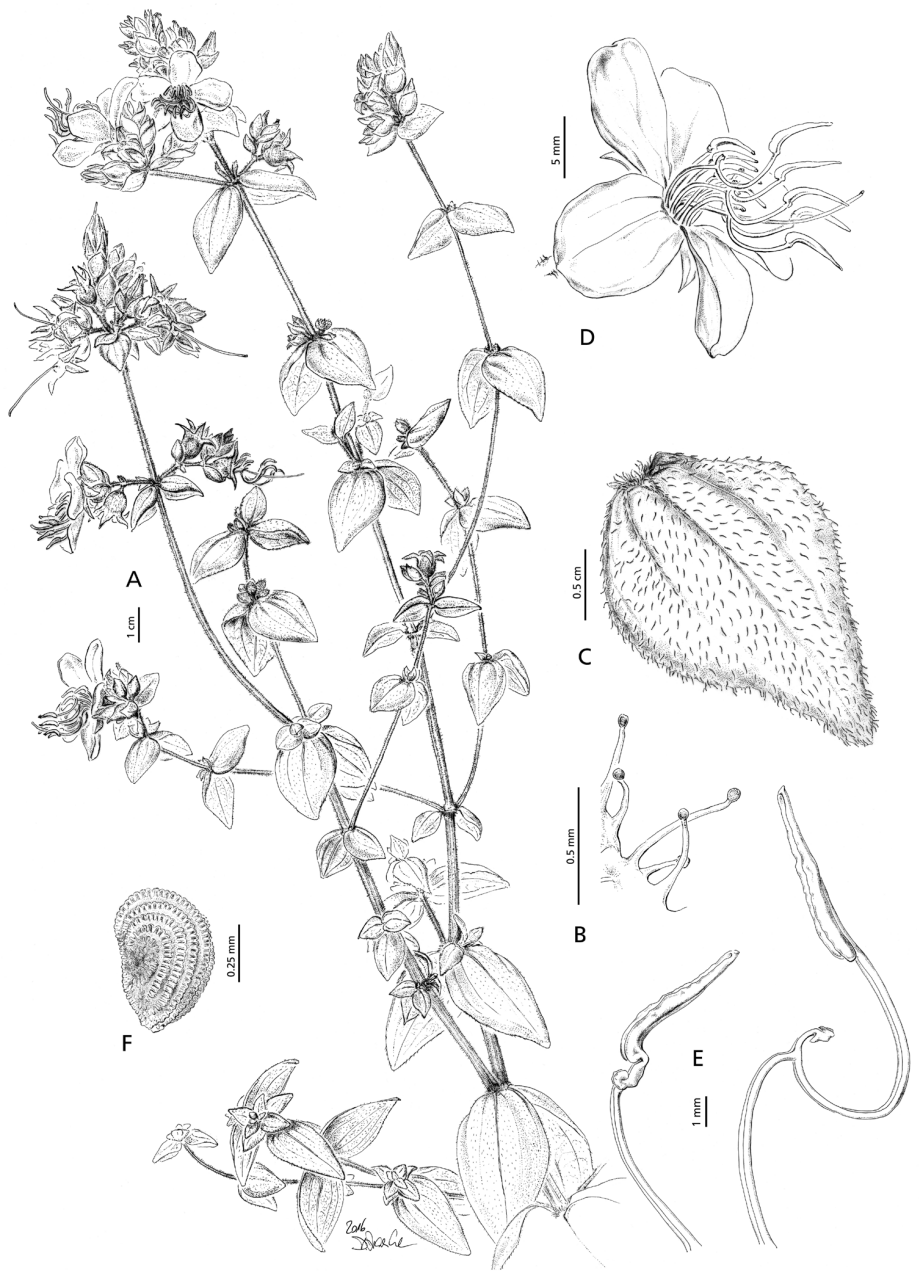
*Argyrella richardsiae*, **A** habit **B** glandular trichomes **C** leaf **D** flower **E** stamens: inner stamen (left), outer stamen (right) **F** seed (drawn from *Mrs H.M. Richards & S. Arasululu* 26190 and *Bidgood et al.* 3935). Illustration by Doris Franke.

#### Diagnosis.

The new species differs from all other species of *Argyrella* by having only glandular trichomes throughout the whole plant (versus a mixture of stellate and glandular trichomes in the other species), secondary branches arising at each node and leaves generally pointing downwards. It resembles *A. bambutorum* (Gilg & Ledermann ex Engl.) Veranso-Libalah & G.Kadereit but differs by having leaf-blades with serrulate margins (versus entire margins in *A. bambutorum*) and the primary pair of lateral nerves disappearing half-way between the base and the apex (versus conspicuous lateral nerves percurrent from the base to the apex in *A. bambutorum*). *Argyrella richardsiae* also differs from the widely distributed *A. canescens* and the Angolan endemic *A. angolensis* (Cogn.) Veranso-Libalah & G.Kadereit by having many new branches or buds arising at each node (versus unbranched herb in *A. angolensis* and *A. canescens*) and leaves sessile with amplexicaul bases generally pointing downwards (versus leaves petiolate with rounded to cordate bases and generally pointing upwards in *A. angolensis* and *A. canescens*) and only glandular trichomes on the hypanthium (versus dense stellate and glandular trichomes on the hypanthium of *A. angolensis* and *A. canescens*).

#### Description.

Erect herb up to 1 m tall with branches arising at each node (Fig. 1A); stems quadrangular, covered with glandular trichomes (0.3–0.7 mm) (Fig. 1B). Leaves sessile, broadly ovate, generally pointing downwards; lamina 15–35 × 7–22 mm, covered with sparse glandular trichomes on both surfaces, apex acute, base amplexicaul, margins serrulate; principal nerves 5–7, lateral nerves fading about half-way from the base and never reaching the apex on the adaxial surface but reaching the apex on the abaxial surface (Fig. 1C). Inflorescence a terminal panicle of cymes with 15–25 flowers or axillary with 5–10 flowers (Fig. 1D). Two caducous bracts, 4–6 × 3–5 mm, pink–mauve, covered with glandular trichomes and enclosing the calyx-tube. Calyx-tube campanulate, 2.5–6 mm in diameter, covered with glandular trichomes. Calyx-lobes 5, triangular, 4.5–6 mm long, persistent, margins and dorsal surface covered with glandular trichomes. Petals 5, pink, 9–13 × 7–8 mm, obovate. Stamens 10, markedly unequal, anthers mauve-purple, filaments yellow, pedoconnectives pink-mauve, appendages yellow. Outer stamens 16–18 mm long, anthers 5–7 mm, filaments 5–7 mm, pedoconnective 7–8.5 mm, strongly curved, appendage ventrally tri-cuspidate, 1–2 mm (Fig. 1E). Inner stamens 9–13 mm long, anthers 3.5–5 mm, filaments 4–5 mm, pedoconnective ca. 1.5 mm long, appendage ventrally bilobed, ca. 0.5 mm (Fig. 1E). Style 22–25 mm long, glabrous. Stigma punctate. Fruit a capsule, dehiscent, ca. 6 mm long. Seeds cochleate, ca. 0.6 mm in diameter, numerous (Fig. 1F).

#### Additional specimens examined.

ANGOLA. Huambo province: Longonjo, Lépi, Caála 1700m, 3 August 1940, (fr), *J. Gossweiler 12147* (LISC030751!, LISC030752!, LISC030753!, LISC030754!, LISC030755!, LISC030756!). TANZANIA. Mpanda district: Uruwira-Tabora road, *Brachystegia* woodland, Kambisama river, 1400 m, 30 September 1970 (fl & fr), *Mrs H.M. Richards & S. Arasululu 26190* (K!, BR!); Mlele beekeeping reserve, riverine woodland, Iloba river, 6°47'56"S, 31°37'33"E, 1562 m, 7 May 2004, *N.A*. *Mwangulango 1193* (MO, MJG!).

#### Etymology.

The species epithet is in honour of Mary Alice Eleanor Richards (also known from her collection labels as Mrs H.M. Richards), who collected extensively in Africa from 1951 to 1974 (Polhill and Polhill 2015). Of our new species *Argyrella richardsiae*, she made a collection which is cited above in ‘Additional specimens examined’.

#### Distribution and habitat.

**Figure 2.**
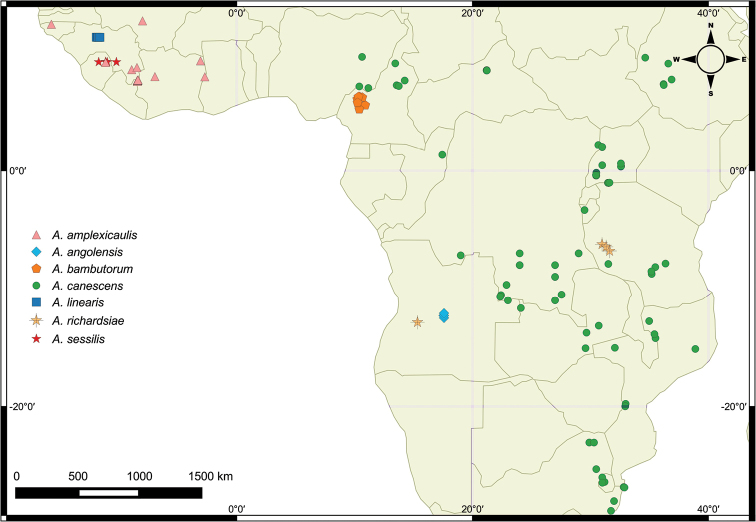
Distribution map of *Argyrella* species.

This species is evidently endemic to the wet miombo woodlands of Mpanda district, southwestern Tanzania and Huambo province, central Angola (Fig. 2). Miombo woodland is a significant biome covering about 10% of the African landmass (White 1983; Campbell et al. 1996, 2007). Miombo woodlands are mainly found in southern and central African countries, and are the dominant vegetation component of Angola, Zambia, Tanzania, Malawi, Mozambique and Zimbabwe (Malmer 2007, White 1983, Campbell et al. 1996, 2007). They are mainly dominated by *Brachystegia* Benth., *Julbernardia* Pellegr. and *Isoberlinia* Craib & Stapf trees of the subfamily Caesalpinioideae, Leguminosae. The wet miombo is found in areas of more than 1000 mm annual rainfall with an elevation of 1000–2500 m. A predominant wet miombo woodland vegetation is composed of riverine woodland along watercourses and marshes in poorly drained and/or low-lying areas, mainly characterised by alluvial soils (Campbell et al. 2007, Lupala et al. 2015). Also, wet miombo has higher tree height (typically > 15 m) and higher floristic diversity which mainly occurs in the northern part of miombo distribution: eastern Angola, northern Zambia, southwestern Tanzania and central Malawi (Frost 1996). It is likely that *A. richardsiae* also occurs in wet miombo woodlands of southern Democratic Republic of Congo (DRC), northern Zambia and southern Malawi and not just disjunctly between Angola and Tanzania. *Argyrella richardsiae* like the other *Argyrella* species grows in marshes.

#### Conservation status.

Proposed IUCN Red List Category: Endangered (EN): B2ab (ii,iii) EOO ~79 km^2^, AOO 32 km^2^. This species is only known from four collections in seepage, marshes or riverine woodlands (wet miombo woodlands) of the Mpanda district, Tanzania and Huambo province of Angola. Although cited on the collection label *Mwangulango 1193* as a locally frequent herb in the Mpanda district, it is still a poorly collected species. As earlier suggested, *A. richardsiae* may also occur in wet miombo woodlands of southern DRC, northern Zambia and southern Malawi, but at the moment we think it is better to treat it as endangered until we are certain that this species is found in other places. Moreover, this species is only known from one collection in Angola since 1940. Generally, miombo woodlands are an important source of livelihood because they provide social, economic, and environmental beneﬁts such as ﬁrewood, timber, medicinal plants, food, and catchment protection, among others. According to Campbell et al. (2007) over 75 million people inhabit areas covered, or formerly covered, by miombo woodland, with an additional 25 million urban dwellers relying on miombo wood or charcoal as a source of energy. As a result, these woodlands have been and are being depleted for the harvesting of timber used for charcoal production, conversion to farmlands and fuel-wood extraction (Campbell et al. 2007, Lupala et al. 2015).

#### GenBank Accession Nos.

KX889285 (ITS), KY248410 (*psbK-psbL*), KY284711 (*accD-psaI*) (see Veranso-Libalah et al. 2017).

#### Discussion.

*Argyrella richardsiae* is similar to *A. bambutorum* but differs by having serrulate leaf margins, intersepalar appendages absent, and stamens dimorphic (versus entire leaf margins, intersepalar appendages present, and stamens isomorphic in *A. bambutorum*). The new species also differs from *A. amplexicaulis*, *A. sessilis* (Hutch. ex Brenan & Keay) Veranso-Libalah & G.Kadereit and *A. angolensis* by its many new branches or buds arising at each node (versus unbranched in *A. amplexicaulis*, *A. sessilis* and *A. angolensis*). *Argyrella richardsiae* has broadly ovate leaves versus lanceolate to linear in *Argyrella canescens* and *A. linearis* (Jacq.-Fél.) Veranso-Libalah & G.Kadereit. Also, *A. richardsiae* has only glandular trichomes on the entire plant and leaves generally pointing downwards versus a mixture of stellate and glandular trichomes with leaves pointing upwards in the other species.

### Key to the species of *Argyrella*

**Table:** 

1	Leaf lamina ovate to broadly ovate, base sessile and amplexicaul or subsessile (petiole < 2 mm long)	**2**
–	Leaf lamina linear or lanceolate-oblong, petiolate (petiole > 2 mm long)	**5**
2	Leaves subsessile; intersepalar appendages present; stamens yellow, subequal in size (isomorphic) (Northwest region of Cameroon)	***A. bambutorum***
–	Leaves sessile to amplexicaul; intersepalar appendages absent; stamens markedly unequal in both size and colour (dimorphic)	**3**
3	Sparingly branched herb; leaf margins entire; principal pair of lateral nerves conspicuous and reaching the apex on the adaxial surface; calyx-tube and stems with stellate-tomentose and glandular trichomes	**4**
–	Much branched herb with buds and branches at each node; leaf margins serrulate; principal pair of lateral nerves fading half-way between the base and the apex on the adaxial surface; calyx-tube and stems with glandular trichomes only (Angola and Tanzania)	***A. richardsiae***
4	Leaf lamina ovate to ovate-lanceolate, < 1 cm wide; calyx-tube with sparse and short stellate trichomes (Guinean region)	***A. amplexicaulis***
–	Leaf lamina broadly ovate, > 1.5 cm wide; calyx-tube with dense stellate-tomentose trichomes (Sierra Leone)	***A. sessilis***
5	Internodes < 2 cm long; leaves linear, the pair of lateral nerves situated very close to the margins; calyx-tube with simple trichomes; intersepalar appendages absent (Guinea)	***A. linearis***
–	Internodes > 2 cm long; leaves oblong-lanceolate, the principal nerves 5–7 in number; calyx-tube with simple setose and short stellate trichomes sometimes mixed with glandular trichomes; intersepalar appendages present	**6**
6	Reticulate secondary venation of the leaves invisible beneath; calyx-tube campanulate, non-glabrescent with age; intersepalar appendages subulate-filiform (Angola)	***A. angolensis***
–	Reticulate secondary venation of the leaves visible beneath; calyx-tube ovoid-subspherical, glabrescent with age; intersepalar appendages shortly subulate or absent (widespread in Africa)	***A. canescens***

## Supplementary Material

XML Treatment for Argyrella richardsiae
